# Validation of the McCluskey Index for Predicting Higher Blood Transfusion in Living Donor Liver Transplantation

**DOI:** 10.7759/cureus.82480

**Published:** 2025-04-17

**Authors:** Joy John, Amal F Sam, Rathnavel G Kanagavelu, Akila Rajakumar, Deepti Sachan, Ashwin Rammohan, Mohamed Rela

**Affiliations:** 1 Department of Liver Anesthesia and Intensive Care, Dr. Rela Institute & Medical Centre, Chennai, IND; 2 Department of Transfusion Medicine, Dr. Rela Institute & Medical Centre, Chennai, IND; 3 Department of Hepato-Pancreato-Biliary (HPB) Surgery and Liver Transplantation, Dr. Rela Institute & Medical Centre, Chennai, IND

**Keywords:** blood transfusion prediction, liver transplantation, massive blood transfusion, mccluskey index, packed red blood cell transfusion

## Abstract

Background

Liver transplantation (LT) surgery is often associated with massive blood transfusion (MBT) due to the complex nature of the procedure and altered coagulation status. Predicting MBT is crucial for effective resource planning and patient management. Existing literature suggests that the McCluskey index has good predictive ability for MBT, although it has primarily been validated in cadaveric transplant programs. This study aimed to validate the index at our center, which mainly performs living donor-related LT (LDLT) using adult-related donors, and identify predictors of blood transfusion in our cohort.

Methods

We retrospectively analyzed data from 533 patients who underwent either cadaveric or LDLT between January 2019 and September 2022. Packed red blood cells (PRBC) were transfused to maintain a target hemoglobin level of 8-9 g/dL. In our study, transfusion of ≥6 PRBC (the 75th percentile) was defined as higher blood transfusion (HBT). A receiver operating characteristic curve was used to assess the McCluskey index’s ability to predict HBT.

Results

Using the threshold of ≥6 PRBC, 32.6% of the study population required HBT. The McCluskey index, which showed an area under the curve (AUC) of 0.82 in the internally validated cohort, yielded an AUC of 0.66 in our cohort. Independent risk factors for HBT identified in our analysis included preoperative hemoglobin level (OR = 0.69, 95% CI: 0.61-0.78), preoperative Model for End-Stage Liver Disease score (OR = 1.71, 95% CI: 1.14-2.58), a history of ICU admission within 60 days prior to transplantation (OR = 1.63, 95% CI: 1.05-2.52), and deceased donor LT (OR = 3.52, 95% CI: 1.1-11.7). A scoring system incorporating these four independent risk factors predicted HBT with an AUC of 0.73, significantly higher than the McCluskey index (p = 0.02).

Conclusions

Most components of the McCluskey index did not appear to be independent risk factors for increased blood transfusion in our cohort, which was predominantly composed of LDLT cases. Nevertheless, as a composite score, the McCluskey index showed some predictive efficacy, though it was inferior to the prediction model based on the independent risk factors identified in our cohort.

## Introduction

Liver transplantation (LT) is widely recognized as the standard treatment for decompensated end-stage liver disease, acute liver failure, nonmetastatic hepatocellular carcinoma, and various other metabolic liver diseases. However, LT surgery is often associated with substantial blood loss and the need for massive blood transfusion (MBT) due to the complex nature of the procedure and the altered coagulation status of patients [1]. Predicting MBT is essential for effective resource planning, including arranging blood products and concentrates, preparing and allocating healthcare personnel, and timing the surgery appropriately.

The McCluskey index has traditionally been used to predict MBT with high accuracy, and it has been externally validated in several studies [2,3]. This index incorporates seven preoperative variables: age >40 years, hemoglobin concentration ≤10.0 g/dL, international normalized ratio (INR) between 1.2 and 1.99, platelet count ≤0.7 lakhs/mm³, creatinine levels >1.1 mg/dL for females and >1.2 mg/dL for males, and repeat transplantation, each of which is assigned a score of one. An INR of ≥2 is assigned a score of 2. The model was internally validated, achieving a high c-statistic value of 0.79. This index was developed in a center where cadaveric LT was predominantly performed [4]. In contrast, living donor-related LT (LDLT) is the predominant form of LT in India and much of the Eastern world [5]. Given this, we conducted this retrospective study at our center to externally validate the McCluskey index in a cohort primarily consisting of LDLT cases.

## Materials and methods

Following approval from the ethics committee, we retrospectively collected data from consecutive patients who underwent cadaveric or LDLT between January 2019 and September 2022. LDLTs were performed only using adult-related donors (up to the fourth degree relative). LT for acute liver failure, combined liver and kidney transplantation, re-transplantation, and split LT were excluded from the analysis.

The collected patient data included age, sex, BMI, etiology, ethnicity, comorbidities, antiplatelet drug use (if applicable), preoperative hemoglobin levels, platelet count, fibrinogen levels, prothrombin time, Model for End-Stage Liver Disease (MELD) score, history of prior spontaneous bacterial peritonitis (SBP), history of prior ICU stay, and the type of transplant (live or deceased donor). Throughout the study period, the core surgical, anesthesiology, and transfusion teams remained consistent, and perioperative anesthetic techniques were standardized.

Anesthesia for all patients was induced with a combination of propofol, midazolam, fentanyl, and atracurium, and maintained with sevoflurane in an oxygen-air mixture. Atracurium and fentanyl infusions were administered during surgery. Hemodynamic monitoring included an arterial line, a 4-lumen 8.5Fr central venous catheter, an 11Fr vascular sheath in the jugular vein for venous access, and a wide-bore peripheral intravenous catheter. A temperature range of 36-37°C was maintained using warming blankets and fluid warmers. Packed red blood cells (PRBC) were transfused to maintain a target hemoglobin level of 8-9 g/dL. Additional blood products were administered based on clinical needs and viscoelastic test assays. No cell saver was used during the study period, and drugs like tranexamic acid or blood products were not administered prophylactically at the start of surgery.

Fluid therapy was titrated based on noninvasive cardiac output monitoring (e.g., pulse pressure variation) in most cases, and invasive monitoring using the PiCCO^®^ system (Pulsion Medical Systems, Feldkirchen, Germany) for selected patients. PiCCO use was indicated for renal dysfunction, hyponatremia, or associated cardiac comorbidities. A second vasopressor was introduced when the dose of noradrenaline exceeded 0.25 µg/kg/min, and vasopressin was universally employed. In our center, ICU admission prior to transplantation for nonsurgical liver disease patients was based on the Modified Early Warning Score (MEWS) [6]. A history of ICU admission, whether in our hospital or another, for nonsurgical reasons within 60 days before the transplant was considered positive.

None of the patients required venovenous bypass, and all deceased donor LTs (DDLT) were performed using the piggy-back technique. Acute kidney injury (AKI) was diagnosed based on the Kidney Disease Improving Global Outcomes criteria [7], and sepsis was defined as a systemic inflammatory response with a positive blood culture [8]. Early allograft dysfunction (EAD) was diagnosed according to Olthoff’s criteria, defined by bilirubin ≥10 mg/dL on postoperative day 7, an INR ≥1.6 on postoperative day 7, and aspartate aminotransferase or alanine transaminase >2,000 IU/L within the first seven days [9]. Mortality rates at 30 and 90 days were also recorded.

MBT was defined as the requirement for ≥10 units of PRBC, while higher blood transfusion (HBT) was defined as the transfusion of ≥6 PRBC, corresponding to the 75th percentile in our data. We analyzed the predictors and outcomes of patients who received HBT and MBT intraoperatively. This study was conducted in accordance with the principles of the Declaration of Helsinki.

For statistical analysis, categorical variables were summarized as frequencies and percentages, while continuous variables were presented as medians with IQRs. The discriminative performance of the McCluskey model was assessed using the receiver operating characteristic (ROC) curve, with the area under the curve (AUC) indicating optimal separation of patients with different outcomes. Independent predictors of HBT in our cohort were identified through binary logistic regression analysis. Variables with p-values <0.05 in the univariate analysis were selected for multivariate analysis. Variables were considered independent risk factors in the final multivariable regression model if the p-value was <0.05. A predictive model for HBT was developed using the regression coefficients of the independent risk factors. Two ROC curves were generated from our cohort: one using the McCluskey model and the other using our own model, the Rela Institute & Medical Centre (RIMC) index. The AUC of both models was compared using the Hanley & McNeil method in MedCalc for Windows, Version 23.0.2 (MedCalc Software, Ostend, Belgium). All other statistical analyses were performed using IBM SPSS Statistics for Windows, Version 23.0 (Released 2015; IBM Corp., Armonk, NY, USA).

## Results

**Figure 1:**
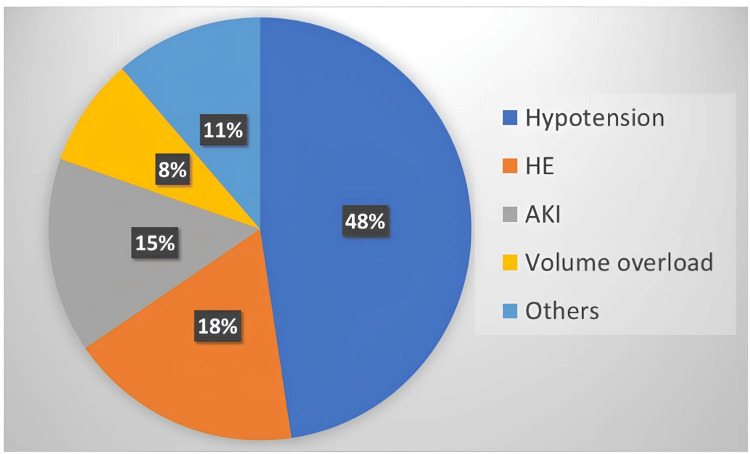
Distribution of presenting complaints among patients admitted to the ICU within 60 days prior to transplantation AKI, acute kidney injury; HE, hepatic encephalopathy

This study included 533 patients, of whom 14 (2.6%) received grafts from deceased donors, while 519 (97.4%) received grafts from living donors. The median MELD score of the study population was 17 (IQR = 13-22), with a median age of 52 years (IQR = 45-59) and a median BMI of 26 kg/m² (IQR = 23-29). The study population consisted of 472 males (80.1%). Chronic kidney disease was present in 26 patients (4.9%). A total of 251 patients (47.1%) had a history of AKI prior to transplantation. Preoperative ICU stay within 60 days before transplantation and a history of prior SBP were reported in 168 (31.5%) and 104 (19.5%) patients, respectively (Table 1). The most common medical reasons for ICU admission prior to LT in our study population were hypotension, followed by encephalopathy (Figure 1).

**Table 1:** Characteristics of the study population AKI, acute kidney injury; CKD, chronic kidney disease; DDLT, deceased donor liver transplantation; HBT, higher blood transfusion; INR, international normalized ratio; MASH, metabolic dysfunction-associated steatohepatitis; MBT, massive blood transfusion; MELD, Model for End-Stage Liver Disease; PRBC, packed red blood cells; SBP, spontaneous bacterial peritonitis

Parameter	N = 533
Age, years	52 (45, 59)
BMI, kg/m²	26 (23, 29)
Gender – Male, n	472 (80.1%)
MELD	17 (13, 22)
Etiology, n	
Ethanol	85 (15.9%)
MASH	135 (25.3%)
Viral	93 (17.4%)
Cryptogenic	59 (11.1%)
Others	161 (30.2%)
Diabetes mellitus, n	251 (47.1%)
Hypertension, n	151 (28.3%)
Incidence of AKI in preoperative period, n	251 (47.1%)
Incidence of CKD, n	26 (4.9%)
Coronary artery disease, n	99 (18.6%)
Patients on antiplatelet drugs, n	35 (6.6%)
History of previous SBP, n	104 (19.5%)
ICU admission within 60 days prior to transplant, n	168 (31.5%)
Type of transplant – DDLT, n	14 (2.6%)
Preoperative hemoglobin, g/dL	9.8 (8.6, 11.2)
Platelets, lakhs/mm³	0.77 (0.54, 1.08)
INR	1.56 (1.23, 2.0)
Serum fibrinogen, g/dL	1.66 (1.25, 2.26)
Serum creatinine, mg/dL	0.88 (0.70, 1.09)
Total bilirubin, mg/dL	1.7 (1.2, 2.9)
Intraoperative PRBC requirement, packs	4 (2, 6)
Intraoperative HBT (use of ≥6 PRBC), n	174 (32.6%)
Intraoperative MBT (use of ≥10 PRBC), n	38 (7.1%)

The median intraoperative requirement for PRBC was four units (IQR = 2-6). Notably, 63 patients (11.8%) did not receive any PRBC transfusions during surgery. To identify factors associated with increased transfusion needs, an analysis was performed using a threshold of ≥6 PRBC, representing the 75th percentile of transfusion volume. Based on this criterion, 174 patients (32.6%) were classified as having received HBT. This cutoff was chosen over the conventional ≥10 PRBC definition for MBT due to the relatively low incidence of MBT in this cohort - only 38 patients (7.1%) required ≥10 PRBC.

**Figure 2:**
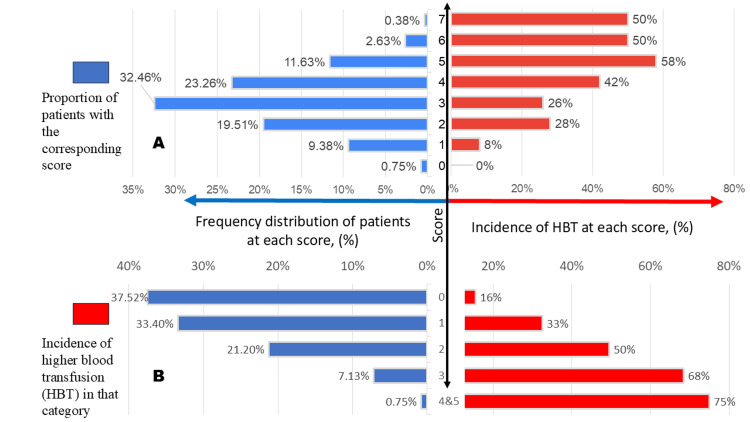
(A) Graph of the McCluskey index; (B) Graph of the comparison model derived from the current study HBT, higher blood transfusion

The calculation and distribution of the McCluskey Index in our cohort are presented in Table 2 and Table 3. A McCluskey score of 5 was associated with a 58% incidence of HBT and an 11.3% incidence of MBT, while a score of ≥6 corresponded to a 50% incidence of HBT and a 6.2% incidence of MBT. An increasing McCluskey score was linked to a higher risk of MBT (Figure 2). The area under the ROC curve (AUROC) for the McCluskey index in predicting HBT and MBT was 0.66 and 0.61, respectively, with an optimal cutoff value identified as ≥4.

**Table 2:** Calculation of the McCluskey index in our cohort INR, international normalized ratio

Parameter	Criteria	Allotted score
Age, years	>40	1
Hemoglobin, g/L	<10.0	1
INR	>2.0	2
	1.2-2.0	1
Platelet count, cells/mL	≤70,000	1
Creatinine, mg/dL	>1.1 for men or >1.2 for women	1
Albumin, g/dL	≤2.4	1
Repeat transplantation		1
Maximum possible McCluskey index = 8		

**Table 3:** Distribution of the McCluskey index in our cohort and the incidence of HBT and MBT at respective scores HBT, higher blood transfusion; MBT, massive blood transfusion; PRBC, packed red blood cells

McCluskey index	Overall incidence (n = 533)	Percentage of HBT (≥6 PRBC), (n = 174)	Percentage of MBT (≥10 PRBC), (n = 38)
0	4 (0.75%)	0%	0%
1	50 (9.4%)	8%	2%
2	104 (19.5%)	28%	6.7%
3	173 (32.5%)	26%	4.6%
4	124 (23.3%)	42%	11.3%
5	62 (11.6%)	58%	11.3%
≥6	16 (3%)	50%	6.2%
p-value		<0.001	0.03

Except for re-transplantation, all factors included in the McCluskey index, along with available clinical variables, were evaluated for their association with HBT using logistic regression analysis. Univariate analysis identified that preoperative hemoglobin, fibrinogen, MELD score, history of AKI, ICU admission within 60 days prior to LT, and DDLT were significantly associated with the need for ≥6 PRBC transfusions (Table 4). Notably, five of the six McCluskey variables - age, INR, platelet count, creatinine, and albumin - did not show significance in the univariate analysis. Preoperative hemoglobin was the only McCluskey variable found to be significant. In the multivariate analysis, four independent predictors of intraoperative transfusion of ≥6 PRBC were identified: preoperative hemoglobin (OR = 0.69; 95% CI: 0.61-0.78), MELD score (OR = 1.71; 95% CI: 1.14-2.58), preoperative ICU stay (OR = 1.63; 95% CI: 1.05-2.52), and DDLT (OR = 3.52; 95% CI: 1.1-11.7). Based on the rounded regression coefficients of these predictors, we assigned scores and developed a comparison model, the RIMC index.

**Table 4:** Logistic regression analysis of the factors associated with transfusion of ≥6 PRBC in our cohort * Level of significance less than 0.05 ** Level of significance less than 0.01 AKI, acute kidney injury; APA, antiplatelet agents; CKD, chronic kidney disease; DDLT, deceased donor liver transplantation; INR, international normalized ratio; MELD, Model for End-Stage Liver Disease; PRBC, packed red blood cells; SBP, spontaneous bacterial peritonitis

Characteristics of patients	Univariate analysis		Multivariate analysis	
	OR	95% CI	OR	95% CI
Age, years	1	(0.97, 1.02)		
Gender (male)	1.36	(0.85, 2.19)		
BMI, kg/m²	1.03	(0.99, 1.07)		
Preoperative hemoglobin, g/dL	0.67**	(0.60, 0.75)	0.69**	(0.61, 0.78)
Preoperative platelet, cells/mL	0.85	(0.62, 1.17)		
Preoperative INR	1	(0.99, 1.01)		
Preoperative fibrinogen, g/dL	0.62**	(0.47, 0.80)	0.99	(0.99, 1)
Preoperative bilirubin, mg/dL	1.01	(0.98, 1.06)		
Pre-op albumin, g/dL	0.89	(0.67, 1.67)		
Preoperative creatinine, mg/dL	0.95	(0.77, 1.16)		
Preoperative AKI	1.86**	(1.29, 2.69)	1.11	(0.73, 1.70)
CKD	2.15	(0.97, 4.74)		
Preoperative MELD	1.09**	(1.06, 1.13)	1.71**	(1.14, 2.58)
Previous SBP	1.96**	(1.27, 3.04)	1.14	(0.68, 1.88)
Preoperative APA therapy	1.08	(0.53, 2.23)		
ICU admissions within 60 days prior to transplant	2.52**	(1.72, 3.70)	1.63*	(1.05, 2.52)
DDLT	3.86*	(1.27, 11.7)	3.52*	(1.1, 11.7)

The comparison model was developed by assigning a score of one to the following criteria: preoperative hemoglobin less than 8.5 g/dL, MELD score greater than 18, and history of ICU admission within 60 days prior to transplantation. The DDLT mode of transplantation was assigned a score of two (Table 5).

**Table 5:** Calculation of an index from the independent predictors in our cohort and the incidence of massive transfusion DDLT, deceased donor liver transplantation; MELD, Model for End-Stage Liver Disease

Parameter	Allotted score
Hemoglobin less than 8.5	1
MELD greater than 18	1
ICU admissions within 60 days prior to transplant	1
DDLT	2

**Figure 3:**
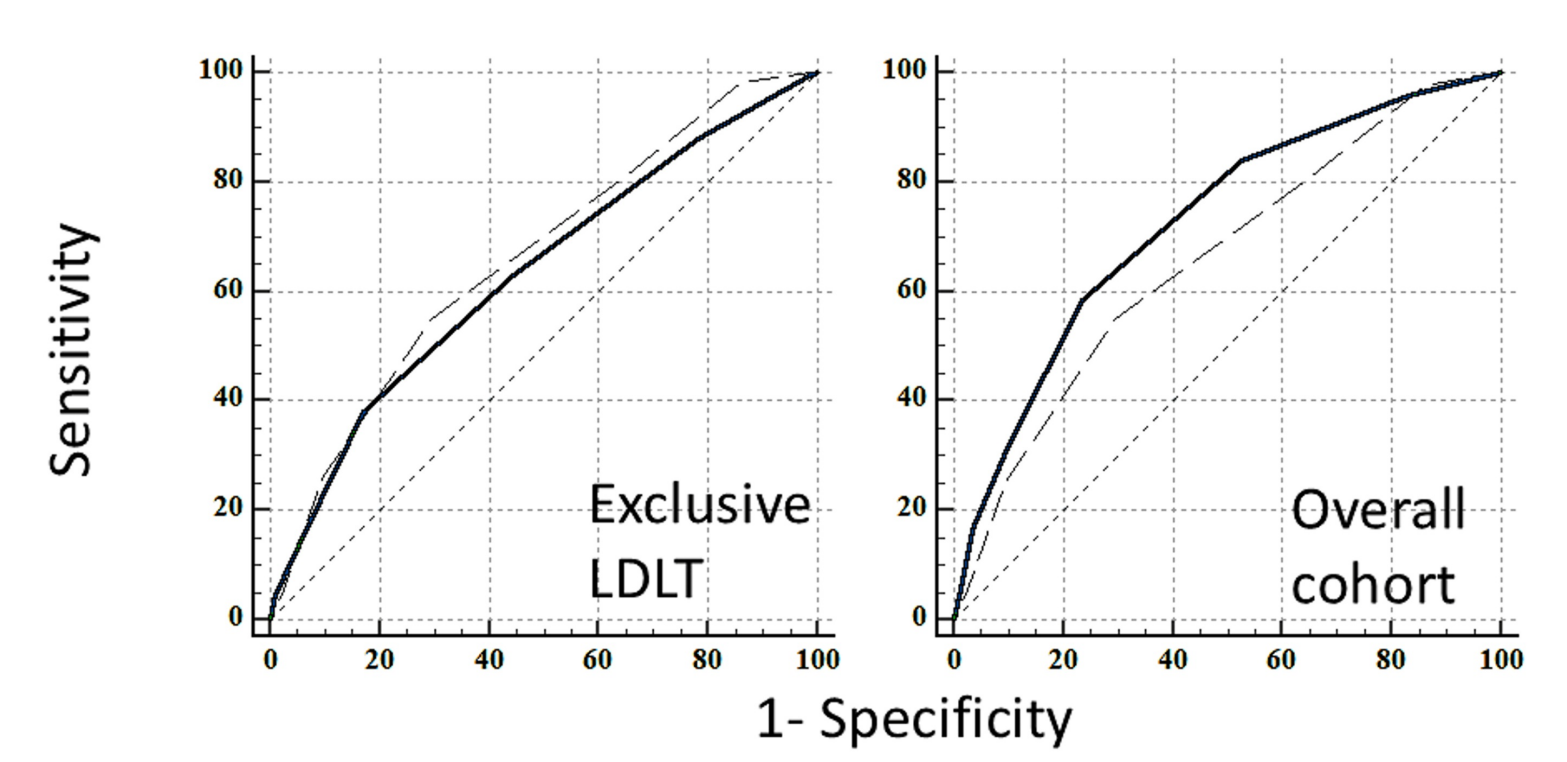
ROC curves of McCluskey index (interrupted line) and the comparison model (continuous line) in predicting ≥6 PRBC during the intraoperative period in both living donor and DDLT (exclusive LDLT and overall cohort) LDLT, living donor-related liver transplantation; PRBC, packed red blood cells; ROC, receiver operating characteristic

In our RIMC model, the total score ranged from 0 to 5, with a score of ≥3 associated with a 73% incidence of HBT (Table 5, Table 6). Of the patients, 37.52% had a score of zero, and the overall incidence decreased as the score increased (Figure 2). Notably, 15% of patients with a score of 0 required intraoperative HBT. As the score increased, the incidence of HBT also rose: 32.50% for score 1, 49.50% for score 2, 68.40% for score 3, and 75% for those with a score ≥4. Our model demonstrated an AUROC of 0.71 in predicting transfusion of ≥6 PRBC in our study group, outperforming the McCluskey index, which had an AUROC of 0.66 (Table 7, Figure 3).

**Table 6:** Distribution of the comparative index derived from the independent predictors in this study and the incidence of HBT and MBT at respective scores HBT, higher blood transfusion; MBT, massive blood transfusion; PRBC, packed red blood cells

Score	Overall incidence (n = 533)	Percentage of HBT (≥6 PRBC), (n = 174)	Percentage of MBT (≥10 PRBC), (n = 38)
0	200 (37.5%)	15.5%	2%
1	178 (33.3%)	32.5%	8.5%
2	113 (21.2%)	49.5%	11%
≥3	42 (7.9%)	73%	16.2%
p-value		<0.001	0.004

**Table 7:** Performance of the two scoring systems in predicting MBTs AUROC, area under the receiver operating characteristic curve; MBT, massive blood transfusion; PRBC, packed red blood cells; RIMC, Rela Institute & Medical Centre

Model		AUROC	Cutoff	Sensitivity	Specificity	Accuracy
To predict ≥6 PRBC	McCluskey index	0.66	≥4	55%	71%	66%
	Comparison index derived from our cohort (RIMC index)	0.71	≥2	49%	81%	71%
To predict ≥10 PRBC	McCluskey index	0.61	≥4	58%	64%	63%
	Comparison index derived from our cohort (RIMC index)	0.67	≥2	47%	73%	71%

We performed a subgroup analysis by excluding DDLT and obtained an ROC curve for 519 LDLT patients. The scores were assigned in the same manner, except for the score of 2 given to DDLT. In this exclusive LDLT cohort, the performance of the McCluskey index remained the same, with an AUC of 0.66 (95% CI: 0.62-0.70). However, our index performed slightly lower than in the DDLT-LDLT mixed cohort (overall cohort), with an AUC of 0.63 (95% CI: 0.59-0.67). The difference in AUC was not statistically significant (p = 0.38) (Table 8, Figure 3).

**Table 8:** Comparison of AUC in ROC curves of the two models AUC, area under the curve; LDLT, living donor-related liver transplantation; RIMC, Rela Institute & Medical Centre; ROC, receiver operating characteristic

Cohort		Overall cohort (n = 533)	Exclusive LDLT cohort (n = 519)
AUC with 95% CI	McCluskey index	0.66 (0.62-0.70)	0.66 (0.62-0.70)
	Our model (RIMC index)	0.73 (0.69-0.76)	0.63 (0.59-0.67)
p-value		0.02	0.38

The incidences of postoperative sepsis and EAD were similar across the groups. The incidence of AKI was 50.9% in patients who received ≥6 PRBC compared to 37.7% in those who did not (p = 0.02), and 50.4% in those who received ≥10 PRBC compared to 38.8% in those who received less (p = 0.02). The median ICU stay was seven days (IQR, 5-11 days) in the MBT and HBT groups, and 6 days (IQR, 5-7 days; p < 0.001) in the non-MBT and non-HBT groups. The hospital stay was longer in patients who received MBT (18 (13, 25) vs. 15 (13, 19); p = 0.007). Mortality at 30 days was similar across all groups. However, at 90 days, 6.8% of patients who received MBT intraoperatively died, compared to 1.7% of patients who did not receive MBT (p = 0.01) (Table 9).

**Table 9:** Outcomes across groups AKI, acute kidney injury; EAD, early allograft dysfunction; HBT, higher blood transfusion; MBT, massive blood transfusion; PRBC, packed red blood cells

Outcome	Transfusion of ≥6 PRBC (HBT)		p-value	Transfusion of ≥10 PRBC (MBT)		p-value
	No (n = 359)	Yes (n = 174)		No (n = 495)	Yes (n = 38)	
EAD, n	23 (6.4%)	16 (9.1%)	0.37	31 (6.3%)	4 (10.9%)	0.17
Sepsis, n	32 (8.9%)	20 (11.4%)	0.78	38 (7.7%)	10 (26.3%)	0.08
AKI, n	135 (37.7%)	89 (50.9%)	0.02	193 (38.8%)	20 (53.4%)	0.02
ICU stay, days	6 (5, 7)	7 (5,11)	<0.001	6 (5, 7)	7 (5,12)	<0.001
Hospital stay, days	15 (13, 19)	16 (13,22)	0.1	15 (13, 19)	18 (13, 25)	0.007
30-day mortality, n	6 (1.52%)	7 (3.72%)	0.43	8 (1.67%)	1 (2.73%)	0.54
90-day mortality, n	6 (1.52%)	10 (5.45%)	0.33	8 (1.7%)	3 (6.8%)	0.01

## Discussion

The use of increased PRBC during LT has been linked to several negative outcomes, including AKI, surgical site infections, and, in some cases, reduced survival of the transplanted organ [10,11]. Numerous studies have analyzed the incidence and associated risk factors to better anticipate such situations. Patient-related factors, such as preoperative hemoglobin, coagulation tests, and the presence of ascites, have been reported as associated with MBT in previous studies [12,13]. Additionally, a history of abdominal surgery has also been linked to MBT [14]. Surgical factors, such as the use of the piggyback technique and venovenous bypass, have also been identified as contributing factors to MBT [15,16].

Several models have been developed to improve the prediction of MBT [17]. The McCluskey risk index for MBT includes seven preoperative variables: age, preoperative hemoglobin concentration, prothrombin time (INR), platelet count, creatinine, and repeat transplantation. This model was internally validated, with a high c-statistic of 0.79. It was also externally validated and reported to perform well [18]. These prediction models could help manage resources more effectively and reduce complications after transplantation. Additionally, recent research has proposed methods to minimize blood loss during LT [19], which would be facilitated by a reliable tool for estimating blood loss.

The existing MBT prediction score, the McCluskey index, was derived from a study in which LDLT accounted for 16% of LT in 2004. In our study, however, LDLT accounted for 97.3% of all LT. In the original study, 22 patients underwent re-transplantation, accounting for 4.7% of the sample. In contrast, the incidence of re-transplantation in our study was too low to be included in the analysis. When compared to the McCluskey study population, our cohort had a slightly lower incidence of MBT (32.6% vs. 41.9%) and a lower median PRBC requirement (4 (2, 6) vs. 5 (3, 9)). While the significance of these differences remains uncertain, they may be attributed to the higher proportion of LDLT patients in our study and/or improvements in surgical techniques over the last two decades [20]. Additionally, the AUROC of the McCluskey index for predicting ≥6 PRBC was 0.82 in the original study, whereas in our population, the AUROC was 0.66. This reinforces the need to modify the LDLT score.

In our study, DDLT recipients demonstrated a higher median PRBC requirement than LDLT recipients. This may be attributed to prolonged cold ischemia time (CIT) and the intense reperfusion changes associated with DDLT compared to LDLT (CIT of 105 minutes in our study group), which can result in coagulopathy and blood loss. Preoperative hemoglobin, preoperative MELD score, and prior ICU admission were additional preoperative factors associated with increased PRBC transfusion requirements. As expected, low initial hemoglobin levels correlated with higher transfusion rates during the intraoperative period. Elevated MELD scores and ICU admissions indicated more severe disease, and patients with severe disease required more intraoperative transfusions. These findings align with the existing literature demonstrating a positive correlation between low hemoglobin levels, higher MELD scores, and increased transfusion requirements during LT [21,22]. Furthermore, we identified prior ICU admissions within 60 days prior to LT as an independent predictor of higher transfusion requirements, alongside MELD and preoperative hemoglobin levels.

Preoperative serum creatinine level was a predictor in the original McCluskey index and in studies that externally validated the index. However, in our study, it was not statistically significant in univariate regression analysis. This discrepancy may be due to the predominance of LDLT in our study, whereas the earlier studies primarily involved DDLT. In LDLT procedures, the ability to schedule surgery allows for better optimization of the patient's condition, in contrast to DDLT. This temporal advantage may have mitigated the impact of creatinine levels.

In a study conducted by Justo et al. [23], albumin, a component of the original score, was not found to be a predictor of MBT. However, they also reported that sodium levels below 137 mEq/L were associated with MBT. Additionally, they found that donation after cardiac death was an independent factor for MBT, which partially supports our finding that DDLT can involve more severe reperfusion and coagulopathy changes.

A primary contradictory finding in our study, compared to the original study and external validation studies, was the absence of an association between conventional coagulation tests and MBT. Fibrinogen, which is often the first factor to reach critical levels in either hemodilution or massive transfusion situations [24], was significant in univariate regression analysis but was not an independent predictor in the final analysis. Other factors, including platelet count and INR, were not associated with MBT. Although platelet count is reduced in cirrhosis, platelet function is enhanced due to compensation by the highly active von Willebrand factor [25]. Moreover, the INR, derived from prothrombin time, is often inadequate for reflecting coagulation status in chronic liver disease due to the rebalancing of hemostasis [25,26]. These factors may explain why conventional coagulation tests were not associated with MBT in our study. The use of viscoelastic point-of-care tests could provide additional insights for future research.

Based on the results of this study, we developed a novel model (RIMC index) for comparison with the McCluskey index, incorporating preoperative factors independently associated with HBT, specifically preoperative hemoglobin level, MELD, ICU admission within 60 days prior to transplantation, and DDLT mode of transplant. Each factor was assigned a score based on its regression coefficient, except for one factor. Our index predicted HBT and MBT with an AUROC of 0.73 and 0.68, respectively. A score of ≥2 indicated a 49.5% probability of intraoperative HBT, while a score of ≥3 indicated a 16.2% probability of intraoperative MBT. Even in the LDLT subgroup analysis, the three-factor scoring system derived from our cohort performed comparably to the six-factor McCluskey index (excluding the “repeat transplantation” component).

Patients who required HBT or MBT had a higher incidence of AKI and a longer median ICU stay postoperatively. Those undergoing MBT also experienced increased hospital stays and 90-day mortality. This association may be direct, or high preoperative MELD may have served as a confounding variable for the increased likelihood of MBT and postoperative mortality. Consequently, using a preoperative risk index could help identify high-risk patients and guide strategies for managing HBTs. These strategies may include ensuring adequate vascular access, more frequent viscoelastic assays, rapid interventions, administration of terlipressin or octreotide to reduce portal pressure and collateral bleeding, the use of clotting factor concentrates, and/or implementation of cell salvage techniques [27-29].

The primary limitation of our study is its retrospective nature, which may have omitted potentially significant preoperative variables, such as the presence of portal vein thrombus and prior abdominal surgery. We used the MELD score as an indicator of disease severity, but the extent of portal hypertension and portal collaterals could not be accurately assessed. Since this was a single-center study, the observation that the McCluskey index performed worse than our locally developed index cannot be generalized. Further studies in the LDLT setting are necessary. A challenge in replicating this scoring system is assigning a score to recent ICU/high-dependency care unit (HDU) admissions within 60 days prior to transplantation. Our institution lacks an HDU, and all patients were admitted to the same ICU. Our center uses the MEWS for patient admission, and other centers may have different admission criteria for wards, HDU, and ICU. Another limitation is that we were unable to collect and analyze thromboelastographic variables due to missing values and inconsistencies. Our center has initiated a study analyzing baseline thromboelastometric values obtained on the day of surgery, prior to transplantation, for their association with blood transfusion. This could provide further insights into predicting blood transfusion requirements.

## Conclusions

Most components of the McCluskey index did not appear to be independent risk factors for HBT in our cohort, where LDLT predominated. However, as a composite score, the McCluskey index demonstrated some efficiency, although it was less effective compared to the prediction model we developed based on independent risk factors from our cohort. The independent risk factors identified in our study were low preoperative hemoglobin, high MELD score, ICU stay within 60 days prior to transplant, and deceased donor-based transplant.
